# Teashirt and C-Terminal Binding Protein Interact to Regulate *Drosophila* Eye Development

**DOI:** 10.3390/genes16091045

**Published:** 2025-09-05

**Authors:** Surya Jyoti Banerjee, Jennifer Curtiss, Chase Drucker, Harley Hines

**Affiliations:** 1 Department of Biological Sciences, Texas Tech University, Lubbock, TX 79409, USA; cdrucker@ttu.edu; 2 Department of Biology, New Mexico State University, Las Cruces, NM 88003, USA; curtij01@nmsu.edu; 3 Department of Biological Sciences, Arkansas Tech University, Russellville, AR 72801, USA; hinesharley19@gmail.com

**Keywords:** *Drosophila*, eye development, teashirt, C-terminal binding protein, CRISPR-Cas9, BAC recombineering, genetic interaction, co-immunoprecipitation, GST pulldown

## Abstract

**Background and Objectives**: The *Drosophila* retinal determination network comprises the transcription factor Teashirt (Tsh) and the transcription co-regulator C-terminal Binding Protein (CtBP), both of which are essential for normal adult eye development. Both Tsh and CtBP show a pattern of co-expression in the proliferating cells anterior to the morphogenetic furrow that demarcates the boundary between the anteriorly placed proliferating eye precursor cells and the posteriorly placed differentiating photoreceptor cells in the larval eye-precursor tissue, the eye–antennal disc. The disc ultimately develops into the adult compound eyes, antenna, and other head structures. Both Tsh and CtBP were found to interact genetically during ectopic eye formation in *Drosophila*, and both were present in molecular complexes purified from gut and cultured cells. However, it remained unknown whether Tsh and CtBP molecules could interact in the eye–antennal discs and elicit an effect on eye development. The present study answers these questions. **Methods**: 5′ GFP-tagging of the *tsh* gene in the *Drosophila* genome and 5′ FLAG-tagging of the *ctbp* gene were accomplished by the CRISPR-Cas9 and BAC recombineering methods, respectively, to produce GFP-Tsh- and FLAG-CtBP-fused proteins in specific transgenic *Drosophila* strains. Verification of these proteins’ expression in the larval eye–antennal discs was performed by immunohistological staining and confocal microscopy. Genetic screening was performed to establish functional interaction between Tsh and CtBP during eye development. Scanning Electron Microscopy was performed to image the adult eye structure. Co-immunoprecipitation and GST pulldown assays were performed to show that Tsh and CtBP interact in the cells of the third instar eye–antennal discs. **Results**: This study reveals that Tsh and CtBP interact genetically and physically in the *Drosophila* third instar larval eye–antennal disc to regulate adult eye development. This interaction is likely to limit the population of the eye precursor cells in the larval eye disc of *Drosophila*. **Conclusions**: The relative abundance of Tsh and CtBP in the third instar larval eye–antennal disc can dictate the outcome of their interaction on the *Drosophila* eye formation.

## 1. Introduction

The vertebrate’s camera-like eye and the insect’s compound eye, such as the eyes of *Drosophila* (fruit fly), are morphologically very different; however, the cellular and molecular mechanisms underlying eye development in them are highly conserved. In *Drosophila*, a retinal determination gene network (RDGN), comprising conserved transcription regulatory molecules with specific spatial and temporal expression patterns in the larval eye precursor tissue, the eye–antennal disc, controls the development of the adult eye, antenna, and other head structures [1,2,3,4,5,6]. The two larval eye–antennal discs produce the two compound adult eyes. Each eye–antennal disc is made of a two-layered epithelium. The epithelium makes a pseudo-sack-like structure, with a columnar epithelium, called the eye disc proper, that develops into the adult eyes, and an overlying squamous epithelium, the peripodial membrane, which forms the head capsule [7,8,9,10,11]. The cells of the eye disc proliferate to produce sufficient eye progenitor cells till the second instar stage. A morphogenetic furrow (**MF**) (a dorsal-ventral groove) originates at the posterior boundary of the disc proper during the third instar stage, and it propagates towards the anterior end of the eye disc. The eye precursor cells, far anterior to the MF, continue to divide asynchronously till late larval life. The anterior cells just ahead of the MF divide twice synchronously and commit to become retinal tissue (i.e., enter the preproneural state), while cells posterior to the furrow differentiate to become photoreceptors [12,13,14,15]. After metamorphosis, these two larval eye primordia tissues produce the two adult compound eyes. Each compound eye is composed of approximately 750 ommatidia, which are arranged in regular hexagonal arrays. Each ommatidium is made of 8 photoreceptor cells and 11 accessory cells [9,15,16,17,18].

The RDGN regulates cell proliferation and retinal specification in the eye disc, making it a valuable experimental model for studying the molecular mechanisms of organ development. A mutation in, or misexpression of, the RDGN members changes the eye developmental process and the structure of the eye–antennal disc, which eventually impacts adult eye development [3,7,19,20,21,22,23,24,25,26]. Moreover, tightly regulated spatial and temporal interactions among the RDGN members control differential gene expression, thereby deciding eye precursor cells’ fate within the eye disc for proper eye development [1,2,5,6,22,27,28]. Hence, understanding the mechanisms underlying these molecular interactions is crucial, particularly for developing treatments for human congenital eye disorders such as aniridia and blindness.

For example, the master transcription factor Eyeless (Ey) is a *Drosophila* RDGN top hierarchy member that is orthologous to the vertebrate PAX6 protein. During the first two larval instars, *ey* transcripts and Ey protein are detected in the eye progenitor cells of the entire eye–antennal discs [8,29,30,31]. After morphogenetic furrow initiation, *ey* expression is detected anterior to the MF, where cells are still proliferating, but is downregulated in cells at the MF, and in the differentiating cells posterior to the furrow [20,29]. Homozygous *ey* mutant flies have smaller eye discs due to the increased death of the eye progenitor cells. The mutants have very small or no adult eyes. Conversely, ectopic expression of *ey* induces eye formation in the fly leg, wing, or antenna [20,21,22,25,29,31,32]. Ey induces cell proliferation and retinal cell-fate determination [20,33,34,35]. However, Ey interacts with other molecular partners to perform these roles during eye development [20,22,26].

Two conserved RDGN members—namely Teashirt (Tsh), a zinc finger transcription factor, and the C-terminal Binding Protein (CtBP), a transcription coregulator—interact with Ey and are required for normal eye development [7,20,22,23,36,37]. The mammalian ortholog of *Drosophila* Tsh, Teashirt Zinc Finger Homeobox Family Member 3 (TSHZ3), plays important roles in the development of smooth muscle of the ureter, neurons that contribute to rhythmic respiration, and cortical projection neurons [38,39,40]. Vertebrates have two orthologs of *Drosophila* CtBP, CtBP1 and CtBP2, which act as antagonists of apoptosis and inducer of tumorigenesis in a context-dependent manner [41]. In *Drosophila*, Tsh and CtBP were initially studied separately for their roles in specifying embryonic segmentation [42,43,44]. Studies suggest that Ey, Tsh, and CtBP show co-expression in the proliferating cells anterior to the morphogenetic furrow of the third instar eye disc, making them potential interactors during eye development [19,20,45,46].

Expression of *tsh* begins in the disc proper epithelium of the first instar eye disc [47]. Its expression in the third instar eye disc is detected in the proliferating precursor cells anterior to the MF [19,20]. It plays different roles depending on the partner molecules present in transcription regulatory complexes. For example, ectopic Tsh requires Homothorax (Hth), a homeodomain protein, and Ey to act together as an ectopic eye inducer in the antennal region of the adult *Drosophila* [20,23,47,48,49]. Its transient and restricted expression in the proliferating cells anterior to the MF of the third instar larval eye disc is likely to induce eye-specific gene expression [7]. Tsh promotes the proliferation of the undifferentiated eye precursor cells anterior to the morphogenetic furrow, along with Ey and Hth, along the dorsal margin of the eye. Conversely, Tsh represses eye development along the ventral margin with Homothorax [7,20,47]. In addition to their genetic interaction and the subsequent effect on eye development, Ey and Tsh were co-immunoprecipitated from the third instar eye–antennal disc lysate, providing evidence of their molecular interaction [20].

Unlike Tsh, CtBP lacks a known DNA-binding domain; thus, it is often recruited by DNA-binding transcription factors [50]. The *Drosophila ctbp* gene produces a 50 kDa and a 42 kDA protein isoform. It is expressed anterior to the MF, both in the proliferating eye precursor cells and in antennal cells of the third instar eye disc [45]. CtBP acts as a transcriptional co-activator or co-repressor in a context-dependent manner [36]. CtBP acts as a linker between chromatin remodeling molecules and transcription factors. It binds directly with transcription factors via their PXDLS (where “X” is any amino acid) motif, both in humans and flies, and regulates different organ development in flies [22,28,36,41,42,45,51,52,53,54,55,56,57,58,59,60,61,62,63]. A LoF mutation in *ctbp* induces cell division ahead of MF, ultimately producing larger adult eyes. Conversely, its overexpression by the *eyless-Gal4* driver results in smaller eyes. These results suggest that the wild-type amount of CtBP is crucial in limiting the division of eye precursor cells and regulating eye differentiation in the eye disc [22,62]. Interestingly, the smaller eye phenotype due to CtBP overexpression has been rescued by a hypomorphic allele of *ey*. Additionally, the small eye phenotype found in flies overexpressing *ey* by the *eyeless-Gal4* driver can be rescued by adding a *ctbp* null allele. Moreover, Ey and CtBP have been immunoprecipitated from a lysate made from eye–antennal discs, suggesting that they interact at the genetic and molecular levels during eye development [22]. However, multiple trials by GST pulldown assays failed to demonstrate a direct binding between CtBP and Ey [22], suggesting a mediator molecule, such as Tsh, may act as a linker between Ey and CtBP. Another study has indicated that Tsh-induced retinal cell proliferation depends on its interaction with CtBP [41]. Hence, molecular interaction between the Tsh and CtBP was also tested by co-immunoprecipitation using a lysate prepared from Kc167 cells [45] and by GST pulldown assay [63]. The positive results from these experiments [45,63] enhanced the likelihood that Tsh and CtBP interact directly in the eye–antennal disc and regulate eye development.

Overall, Ey, Tsh, and CtBP showed co-expression in the proliferating cells anterior to the MF of the third instar eye disc; Tsh and CtBP were shown to interact with Ey in separate experiments to regulate *Drosophila* eye development; and Tsh was bound with CtBP in a cell line [20,22,45]. Thus, we hypothesized that Tsh and CtBP interact genetically through direct binding at the molecular level in the *Drosophila* eye disc to regulate adult eye development. In this study, we generated a transgenic fly strain by tagging the genomic tsh gene with GFP using the CRISPR-Cas9 technique and produced another transgenic fly strain by incorporating the FLAG-ctbp gene into its genome via the BAC recombineering technique. We confirm functional expression of the EGFP-Tsh and FLAG-CtBP proteins in these strains. Furthermore, we confirm that Ey, Tsh, and CtBP have overlapping expression patterns in the proliferating cells anterior to the morphogenetic furrow (MF) of the third instar larval eye–antennal disc. Our data reveal that overexpression of *tsh* by the *eyGal4* driver stunted the development of the larval eye disc and adult eye. On the contrary, knocking down *tsh* expression by the *eyGal4* driver showed no impact on the adult eye. We also report that up- and downregulation of *ctbp* expression by the *eyGal4* driver produced smaller and slightly larger adult eyes, respectively. Furthermore, we provide genetic and molecular evidence to demonstrate that Tsh and CtBP bind directly in the eye disc, and their interaction affects adult eye development. In summary, our findings strengthen the theory that CtBP associates with Tsh, and together they contribute to *Drosophila* eye development in a dosage-dependent manner.

## 2. Materials and Methods

### 2.1. Drosophila melanogaster Fly Strains and Fly Husbandry

Abbreviation of *IR* in superscript represents respective RNAi (RNA interference) strains. 

Sp/Cyo = (Sternopleural mutation and balancer with curly mutation gene on the second chromosome) to make stock fly strains of EGFP-Tsh/ Cyo (CRISPR-Cas9 generated) (available in the lab).

*EGFP-tsh* and *FLAG-ctbp* transgenic flies are generated in this project as described in detail in Section 2.

R. Chen kindly gifted the *eyeless-FLAG* fly line. The eyeless gene was tagged at the 3′ end (C-terminus of the eyeless protein) and inserted in the 68A region of the genomic chromosome, which could rescue the eyeless loss-of-function mutant phenotype [22].

K. Cadigan kindly gifted the *P[GSV]^A396^* fly line. This transgenic fly had a bidirectional EP element inserted into the first intron of *ctbp* (i.e., *UAS-ctbp*) [22].

*UAS-ctbp ORF* (flyorf.ch F001272) fly was obtained from the FlyORF (Zurich ORFeome Project).

Flies obtained from the Bloomington Drosophila Stock Center are listed below with the stock number (Bl#).

Bl# 52669 = y [1] M{vas-Cas9.S}ZH-2A w [1118] = Flies having Cas9 expression under the “vas” promoter at the embryonic stage to generate transgenic flies (Rainbow Transgenics).

Bl# 5535= w[*]; P{w[+m*] = GAL4-ey.H}4-8/CyO = *eyeGal4/CyO.*

Bl# 52216 = w[*]; P{w[+mC] = UAS-tsh.G}2 = *UAS-tsh.*

Bl# 31334= y [1] v [1]; P{y[+t7.7] v[+t1.8] = TRiP.JF01291}attP2/TM3, Ser [1] = *UAS-ctbp^IR^*.

Bl# 28022 = y [1] v [1]; P{y[+t7.7] v[+t1.8] = TRiP.JF02856}attP2 = *UAS-tsh^IR^.*

Bl# 50763 = tsh [8]/CyO P{ry[+t7.2] = en1}wg[en11] = *tsh^8^/ CyO*, (loss of function *tsh* allele, homozygous lethal).

Bl# 4776 = w [1118]; P{w[+mC] = UAS-GFP.nls}8 = *UAS-GFP* (with nuclear localization signal).

Flies were cultured and maintained in a standard *Drosophila* fly incubator with a set temperature of 25 °C. All fly crosses were set at 25 °C. Flies were cultured in vials with freshly prepared food using the Bloomington fly food formula.

### 2.2. In Vivo EGFP Tagging at the 5′ of the Genomic Tsh Gene by the CRISPR-Cas9 Method to Generate N-Terminal EGFP-Tsh Fusion Protein

We tagged the genomic *tsh* gene by inserting the EGFP sequence at the 5′ end using CRISPR-Cas9 as described below (modified from [64,65]). We used the online tool (http://targetfinder.flycrispr.neuro.brown.edu/; last accessed on 9 July 2025) and selected two unique guide DNA sequences (the target sequence for gDNA1: 5′ GCTTCTTTGACTAGATATGATGG 3′, and the target sequence for gDNA2: 5′ GGCGCTGAATTTATAACGACAGG 3′), which were the closest upstream and downstream DNA sequences to the first codon (ATG) in the *tsh* gene. The gDNA 1 and 2 (both sense and antisense oligonucleotides) were ordered from the company Integrated DNA Technologies (Coralville, IA, USA). For each guide DNA, sense and antisense strands were ligated to produce double-stranded gDNA (ds gDNA) following the exact protocol available online (https://flycrispr.org/; last accessed on 9 July 2025). Each ds gDNA was cloned separately in the pU6-BbsI-gRNA plasmid under the U6 promoter following the exact protocol (https://flycrispr.org/; last accessed on 9 July 2025). The U6 promoter is part of the spliceosome gene U6; therefore, the DNA construct under this promoter is supposed to be transcribed in all eukaryotic cells. Next, the entire DNA sequence from just upstream of ATG in the *tsh* gene to the flanking 1 kb upstream of the gDNA1 was PCR-amplified using fly genomic DNA as a template and purified using a PCR purification kit from Qiagen. The PCR-purified clone1 DNA sequence was then inserted upstream of an EGFP-carrying vector (pUAS_N_EGFP_BD_ATTB, Catalog# 1505, Drosophila Genomics Resource Center) by the infusion cloning method. Because the EGFP coding DNA sequence already had its own start codon (ATG), thus the ATG was not included in the clone1 DNA sequence. Successful cloning was verified by restriction digestion and sequencing. Likewise, the clone2 DNA sequence, which contained gDNA2 plus 1000 base pairs downstream of it, was inserted downstream of the EGFP sequence in the vector containing clone1 by similar techniques and verified by sequencing as well. All three vectors were prepared using a Qiagen midiprep kit to retrieve good-quality and high-concentration plasmids. The three plasmids were sent to Rainbow Transgenics (Rancho Cucamonga, CA, USA), a company that subsequently injected all three plasmids into the 52669 transgenic fly embryos containing Cas9 under the Vas promoter (expressed during embryonic life) in their genome. Therefore, the Cas9 enzyme, guided by the presence of guide RNAs transcribed from the pU6-BbsI-gRNA produced in these embryos, would cleave double-stranded DNA near the PAM sequence in the fly genome. The entire DNA sequence containing EGFP flanked by the two 1 kb homologous DNA sequences would serve as the template during homology-directed repair of the genome, which would incorporate the EGFP DNA upstream of the *tsh* gene (present on chromosome 2). Flies that emerged from these embryos were crossed with each other, and individual offspring in the F2 generation were crossed with Sp/Cyo (Sternopleural mutation and balancer with curly mutation gene on the 2nd chromosome) flies to generate hundreds of stable stocks. After that, genomic DNA was isolated from flies in each stock, and PCR was performed to amplify the region of interest. Depending on PCR size and sequencing, the genomic DNA of the *EGFP-Tsh* transgenic fly strain was sent for sequencing to verify insertion of the exact sequence. After sequence verification, one stock was selected for further analysis.

To confirm that the Tsh protein is functional in the *EGFP-tsh* transgenic flies that should produce EGFP-Tsh fusion protein, we crossed the *EFPF-tsh/ Cyo* flies (had curly wings) with *tsh^8^/Cyo* flies (had curly wings). The *tsh^8^* allele is a non-functional allele of *tsh,* and the homozygous *tsh^8^/tsh^8^* flies are lethal. We found non-curly living flies (*tsh^8^/EGFP-tsh*) from the above cross, suggesting that the EGFP-Tsh protein is functional. We performed PCR to verify the presence of the *EGFP*-*tsh* sequence in the non-curly *tsh^8^/EGFP-tsh* fly strain. The *EGFP-tsh* transgenic stock had no visible mutant phenotype in the adult eye.

### 2.3. In Vivo 5′ FLAG Tagging of the Ctbp Gene by BAC Recombineering to Generate N-Terminal FLAG-CtBP Fusion Protein

The production of the transgenic fly with a 5′ FLAG-tagged *ctbp* gene was performed by BAC recombineering techniques, as described by [66]. Briefly, a specific primer set produced the PCR product for recombineering using the *N*-Flag-4C vector as template (*N*-FLAG-CtBP Forward Primer: CGACGACGAAGAGCAGAGGAC AGCAGCAGACAGATTGAAAAACAGCGAAATGGATTACAAGGATGACGAC, and *N*-FLAG-CtBP Reverse Primer: CCCTTGACATCGATGCGCGAACGCTTCGGCATCATCAGATTTTTGTCACTAGTGGATCCCCTCGAGGGAC). Next, the *Dpn1-*digested PCR product was gel-purified and co-transformed with the P(acman) [i.e., the BAC containing the *ctbp* gene and the Kanamycin-resistant gene] [67] into DY380 cells (containing a plasmid with the chloramphenicol-resistant gene) by electroporation. The transformed colonies were selected on LB-agar media prepared with Kanamycin and Chloramphenicol and grown on LB media containing both antibiotics for subsequent isolation of the P(acman) having the tagged gene (*FLAG-ctbp*). Isolated P(acman) was verified for the proper recombination event by PCR, restriction digestion, and sequencing. The correct P(acman) vector was transformed into SW106 cells and grown in the Chloramphenicol-containing LB media. The Kanamycin cassette was removed by adding arabinose to the LB media that induced Cre. A diluted sample of these cells was plated on Chloramphenicol-containing LB-agar media. Next, P(acman) was isolated and verified by PCR and sequencing for the successful removal of the Kanamycin cassette. This recombinant product was injected into the fly embryos, and the correct progeny was established through a proper selection process. The *FLAG-ctbp* transgenic stock had no visible mutant phenotype in the adult eye.

### 2.4. Immunohistochemistry and Confocal Microscopy

Histological staining of the third instar larval eye–antennal discs was performed following standard protocol. Larval eye discs were dissected in ice-cold PBS (10× Phosphate Buffer Saline recipe: 1.37 M NaCl, 27 mM KCl, 18 mM KH_2_PO_4_ and 100 mM Na_2_HPO_4_ in one Liter of deionized pure water) and fixed using 4% paraformaldehyde in 1× PBS for 20 min at room temperature (RT) with subsequent washing in PBT (0.1% Triton in 1× PBS) and BBT (PBT with 0.1% bovine serum albumin) for three times, each for 5 min. Samples were incubated overnight at 4 °C with primary antibodies diluted in BBT. Primary antibodies used were mouse anti-Elav (DSHB, Iowa, IA, USA) at a 1:250 dilution. and rabbit anti-EGFP (ThermoFisher Sc, Waltham, MA, USA) at a 1:1000 dilution. antibodies. The tissue was washed with BBT and incubated with appropriate secondary antibodies (Jackson ImmunoResearch, West Grove, PA, USA) at a 1:200 dilution. for 2 h at RT. Tissue was washed in PBT and mounted in 80% glycerol mixed with 5% anti-fade (*N*-propyl gallate) on a slide. Images were captured using a confocal microscope (Leica TCS SP5 Laser Scanning Confocal Microscope, Leica Microsystems, Mannheim, Germany). The z-stacks of images were processed and analyzed using Image J [68].

### 2.5. Scanning Electron Microscopy (SEM)

Adult flies were frozen at −80 °C for 15 min to immobilize them. Images of the adult fly’s eyes were taken using a Hitachi TM1000 Tabletop Scanning Electron Microscope (Hitachi High-Tech Corporation, Tokyo, Japan). All images were taken at the same magnification (300X).

### 2.6. Quantification of Ommatidia of Adult Eye SEM Images

We counted the number of ommatidia in adult fly eyes of different strains using the EyeHex program [69]. SEM images of adult eyes were analyzed. Manual segmentation for ommatidia classification was performed on one image per genotype and was subject to training classification along with the remaining images in the sample set to generate a probability map. Hexagonal expansion was then performed on each image individually using the probability maps generated in the previous steps. Following the generation of hexagonal ommatidia, manual corrections were performed on each image individually to correct errors introduced by the EyeHex program. The EyeHex program detected ommatidia that were marked by green circles, the manual corrections were marked by red circles, and the origin of hexagonal expansion was marked by green circles outlined in pink. The average visible ommatidia count was then calculated using all images in a sample set. The data is available in the Supplementary Materials File 1.

### 2.7. Light Microscopy

Adult flies were frozen at −80 °C for 15 min to immobilize them. All images of the adult fly’s eyes were taken using a Leica EZ4 W Stereomicroscope under the same magnification (30X).

### 2.8. Co-Immunoprecipitation (Co-IP)

A total of 250 third instar larval eye–antennal discs from *EGFP-tsh* flies were dissected in chilled 1× PBS and placed into a microfuge tube with 500 µL of chilled 1× PBS. The tube was spun briefly at 10,000× *g* in a pre-chilled (4 °C) centrifuge machine. All the supernatant was carefully removed. The tissue was lysed with 250 µL of RIPA buffer mixed with 1× protease inhibitor cocktail and 1 mM PMSF by gently pipetting 8–10 times. The sample was incubated for another 10 min at room temperature (RT) with vigorous shaking in the tube. Next, the tube was centrifuged for 5 min at 10,000× *g* at 4 °C. All the supernatant was carefully pipetted into a new tube, and any precipitate was discarded. Three microfuge tubes, one with 30 µL supernatant and the other two with equally distributed rest of the supernatant, were prepared. Exactly 30 µL of 2× Laemmli buffer mixed with β-Mercaptoethanol was added to the first tube with 30 µL of lysate, incubated at 65 °C for 10 min, and stored at −80 °C for later analysis. This crude lysate would serve as a positive control. In one of the other two tubes, the rabbit anti-EGFP antibody (1:50) (Cat. No. A-11122, Life Technologies, Coralville, IA, USA) was incubated with the sample in a rotating machine at RT for 1 h, which would serve as the Co-IP experimental sample. After an hour, equilibrated 40 µL Dynabeads Protein-G (Cat. No. 10003D, Invitrogen/Thermo Fisher Sc, Waltham, MA, USA) was added and incubated at RT for another hour. During these two hours, equilibrated 40 µL Dynabeads Protein-G was incubated with the sample in the third tube under the same conditions, which would serve as the negative control. After two hours of incubation, beads in both tubes were washed with ice-cold 1× PBS three times, each for 5 min. After the third wash, the protein was eluted with 40 µL 2× Laemmli buffer mixed with β-Mercaptoethanol and incubated at 65 °C for 10 min. A total of 25 µL of the crude lysate, Co-IP experimental sample, and negative control) were run in a 10% SDS-PAGE denaturing gel and transferred onto a PVDF membrane.

During the Western blot development, the membrane was blocked with TBST [TBS: 20 mM Tris-HCL, 140 mM NaCl, pH 7.5 + 0.1% (v/v) Tween 20] containing 5% dry milk for 1 h at RT on a gentle shaker. The membrane was then washed with TBST three times for 5 min each at RT. The membrane was incubated with the primary rabbit anti-CtBP antibody (1:500) (a kind gift from Arnosti lab) in TBST + 5% dry milk for 2 h at RT. The membrane was then washed with TBST three times for 5 min each at RT. The membrane was incubated with the HRP-conjugated anti-rabbit secondary antibody (Jackson Immunology, West Grove, PA, USA) at a 1:2500 dilution.in TBST + 5% dry milk for 2 h at RT. Next, the membrane was washed with TBST three times for 5 min each at RT. Then, the membrane was washed with TBS three times for 5 min each at RT. Detection was performed with the ECL-based solution, revealing a band approximately 50 kDa in size, indicating the presence of CtBP.

### 2.9. GST Pulldown Assay

GST pulldown assay was performed as described in Banerjee et al. (2024) [28]. Promega’s MagneGST Protein Purification System (Catalog# 17316) was used to perform GST pulldown experiments. The Glutathione S-Transferase (GST) and GST-fusion Bait (GST-Tsh and GST-CtBP) proteins were produced in *Escherichia coli* cells from the cloned constructs in the pGEX 4T-2 vector and purified by Glutathione beads. The biotinylated prey proteins (Tsh and CtBP) were produced from pTNT vectors containing the cloned prey protein coding sequences by in vitro translation using Promega’s TNT t7 quick-coupled IVT (in vitro transcription and translation) system (Catalog# L1170) following the manufacturer’s protocol.

We dissected 80 wild-type third instar eye–antennal discs in 1× PBS (ice-cold) and extracted total RNA using TRIzol (Thermo Fisher Scientific, Waltham, MA, USA). The cDNA was synthesized from the RNA using the iScript Reverse Transcriptase Super mix (Cat. No.1708841, BioRad, Hercules, CA, USA) kit per the manufacturer’s protocol. PCR was performed to produce the *ctbp* and *tsh* coding DNA sequences (CDS) from cDNA. The PCR products were verified by sequencing. Next, we cloned the purified PCR products into pGEX 4T-2 or pTNT vectors by In-Fusion cloning (In-Fusion HD Cloning Plus CE, Cat. No. 638916, Takara Bio/ClonTech) and verified the final product by sequencing.

The *GST*, *GST-ctbp*, and *GST-tsh* bait protein-coding DNA sequences were cloned separately into the pGEX 4T-2 plasmids under the tac promoter. They were transformed into BL21 cells (Cat. No. C2530H, New England Biolabs, Ipswich, MA, USA). Each bait protein’s expression was induced in the BL21 cells by growing them in the Luria Broth media containing 0.5 mM IPTG (Cat. No. I6758, Sigma-Aldrich, St. Louis, MO, USA) for 4 h at 28 °C on an incubator shaker at 250 RPM. Next, the cells were lysed in a solution containing 100 µL Bugbuster (Cat. No. 78243, Invitrogen/Thermo Scientific, Waltham, MA USA), 1 mM PMSF (Cat. No. P7626, Sigma-Aldrich, St. Louis, MO, USA), Protease inhibitor cocktail (Cat. No. 88666, Pierce/Thermo Scientific, Waltham, MA, USA), and 1 µL of DNase (Cat. No. 18068-015, Invitrogen/Life Technologies, Waltham, MA, USA) to isolate the proteins. The Tsh and CtBP prey proteins were expressed independently from their coding sequences, cloned into the pTNT plasmid under the T7 promoter, using the in vitro translation system (IVT). The lysine residues in the prey proteins were biotinylated at the ε-amino group. The expressions of the GST, GST-Tsh, and GST-CtBP proteins were confirmed by Western blots using anti-GST antibody (Cat. No. MA4-004, Thermo Scientific) and HRP-conjugated secondary antibody. Similarly, the IVT Tsh (~105 kDa protein) and CtBP (~42.5 kDa) proteins’ expressions were confirmed by Western blots using Peroxidase−Streptavidin (Cat. No. 016-030-084, Jackson Immuno) antibody.

To perform the GST pulldown experiments, Glutathione beads were equilibrated and resuspended in GST wash buffer. One aliquot of the beads was incubated with 20 µL of GST-tagged bait proteins (either GST-Tsh or GST-CtBP), while another aliquot was incubated with 20 µL of GST alone as a negative control for 30 min at room temperature (RT), followed by three washes with GST wash buffer. Thus, GST or GST-fusion proteins became bound to the Glutathione beads. Subsequently, 20 µL of in vitro synthesized prey protein was added to each aliquot and incubated for an hour at RT with gentle rocking. The combinations of bait and prey proteins used in the assay are listed in Table 1. The mixtures were washed with GST wash buffer three times. Then, 20 µL of each mixture was added with a protein denaturing solution [a 19:1 mixture of 2× Laemmli Sample Buffer (Cat. No. 1610737, BioRad) and β-mercaptoethanol (Cat. No. 444203, Sigma)] and heated at 65 °C for 10 min. The samples were run on an 8% SDS-PAGE gel and transferred to PVDF membranes. Western blotting was performed using Peroxidase−Streptavidin (Cat. No. 016-030-084, Jackson Immuno) Antibody and ECL detection reagents to identify the biotinylated prey proteins (in vitro translated Tsh or CtBP) that were pulled down by the GST or GST-fusion bait proteins (as shown in Table 1).

### 2.10. PCR, Restriction Digestion, Cloning, Plasmid Purification, and Transformation

Primers for PCR were designed using serial cloner software, and the annealing temperature was verified from the NEB (New England Biolab) Tm calculator. Several high-fidelity polymerases were used (e.g., TaKara Taq). Restriction digestions were performed following the manufacturer’s protocol, depending on the enzymes (NEB). Cloning was performed following the In-fusion cloning protocol. Plasmid purification was performed using Qiagen column-based methods. Transformation was performed following the manufacturer’s protocol, depending on the competent cells.

### 2.11. Western Blot

Western blots associated with Co-immunoprecipitation and GST pulldown assays are described in the respective Section 2. Blot images were acquired using a ChemiDoc from BioRad.

## 3. Results

### 3.1. Ey, Tsh, and CtBP Exhibit Overlapping Expression in the Drosophila Larval Eye–Antennal Disc

The transcription factor Tsh and the transcription coregulator CtBP function in the nucleus. Earlier studies have reported that the Tsh protein is expressed in the proliferating cells anterior to the morphogenetic furrow of the *Drosophila* third instar larval eye disc [19,20]. The CtBP protein is expressed in the proliferating cells anterior to the morphogenetic furrow of the eye disc and the antennal disc of the third instar larvae [45].

**Figure 1:**
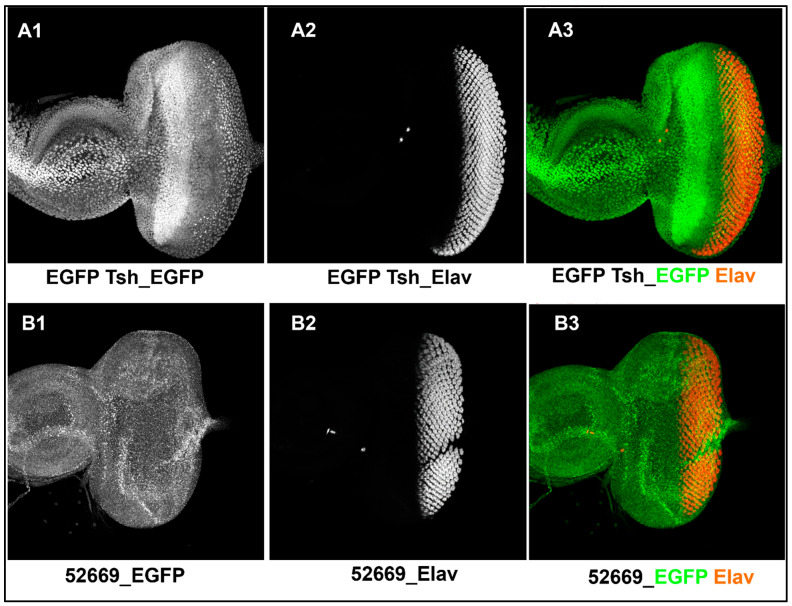
Confocal images of the third instar larval eye–antennal discs showing Tsh and Elav expression patterns (the anterior side is on the left). The top and bottom panels contain the confocal images of the third instar larval eye–antennal discs dissected from the *EGFP-tsh* (**A1**–**A3**) and the BL# 52669 (**B1**–**B3**) (negative control) flies, respectively. The eye–antennal disc from the *EGFP-tsh* fly showed Tsh expression (identified by GFP expression) in the anterior part (to the left) of the eye disc (**A1**), which was absent in the control disc (**B1**). Elav expression was seen at the posterior part of both the eye discs (**A2**,**B2**). In the merged images (**A3**,**B3**), the morphogenetic furrow could be seen as an unstained region between the cells expressing Tsh (green) and Elav (red) in (**A3**).

We first generated transgenic flies with a 5′ *EGFP-*tagged *tsh* (*EGFP-tsh*) gene by CRISPR-Cas9. The transgenic flies produced EGFP-Tsh fusion protein in which Tsh remained functional (see Section 2). Next, we confirmed the Tsh expression pattern in the third instar larval eye–antennal discs of these flies by immunohistological staining with anti-EGFP and anti-Elav antibodies (marker for differentiating photoreceptor posterior to the eye-disc), followed by confocal microscopy. A distinct signal of EGFP elicited by the EGFP-Tsh-fused protein was observed in front of and adjacent to the morphogenetic furrow (MF) of the eye disc (Figure 1(A1,A3)), but not in the eye disc dissected from the parental strain 52669 (used as negative control) (Figure 1(B1,B3)). However, both discs showed Elav expression in the differentiating tissue posterior to the MF (Figure 1(A2,B2) and (A3,B3). Thus, functional Tsh was expressed in *EGFP-tsh* larval eye–antennal discs in its wild-type pattern, as described earlier [19,20].

**Figure 2:**
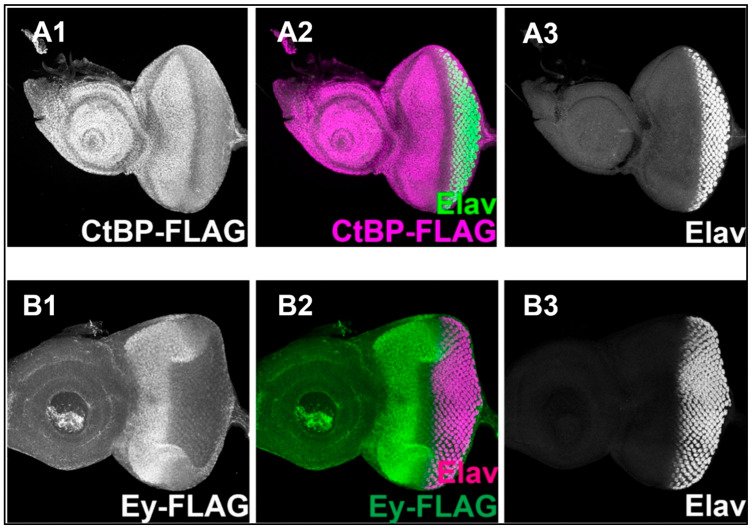
Confocal images of the third instar larval eye–antennal discs showing CtBP, Elav, and Ey expression patterns (the anterior side is on the left). The top and bottom panels contain the confocal images of the third instar larval eye–antennal discs dissected from the *FLAG-ctbp* (shown as CtBP-FLAG) (**A1**–**A3**) and *Ey-FLAG* (positive control) (**B1**–**B3**) flies, respectively. The eye–antennal disc from the *FLAG-ctbp* fly showed CtBP expression (identified by FLAG expression) in the anterior part of the eye disc and the entire antennal disc (**A1**). The eye–antennal disc from the *Ey-FLAG* fly showed Ey expression (identified by FLAG expression) in the anterior part of the eye disc (**B1**). Elav expression was seen at the posterior part of both the eye discs (**A3**,**B3**). In the merged images (**A2**,**B2**), the morphogenetic furrow could be seen as an unstained region between the cells expressing CtBP (pink) and Elav (green) in **A2**; and Ey (green) and Elav (pink) (**B2**).

Similarly, we immunostained the third-instar eye–antennal discs dissected from the *FLAG-ctbp* and *ey-FLAG* transgenic fly stocks with anti-FLAG and anti-Elav antibodies. The confocal image showed strong expression of CtBP as marked by the FLAG expression in the proliferating cells in front of the morphogenetic furrow of the eye disc, and in the cells of the antennal disc (Figure 2(A1)). FLAG signals were also observed in the same pattern as the wild-type Ey expression in the *ey-FLAG* files (positive control) (Figure 2(B1)). Both discs exhibited the wild-type Elav expression pattern (Figure 2(A3,B3)). These results agree with previous findings [20,45].

It was observed that CtBP (Figure 2(A1)) and eyeless (Ey) (Figure 2(B1)) are expressed ubiquitously in all eye precursor cells ahead of the MF, whereas Tsh expression was restricted in fewer rows of cells anterior to the MF (Figure 1(A1,A3)), of the third instar eye disc. Thus, Tsh and CtBP showed co-expression in a fraction of the proliferating eye progenitor cells of the eye disc, making the molecular interaction between them possible in those cells, which can affect the process of eye development.

### 3.2. Tsh and CtBP Interact Genetically During Drosophila Eye Development

Several studies have investigated the role of candidate genes in eye development by altering a gene’s expression levels through up- or downregulating its transcription using the UAS-Gal4 [70,71] and RNA interference (RNAi, *aka* IR) systems, followed by analyzing the resulting adult eye phenotypes. Next, simultaneous manipulation of gene expression levels of two candidate genes, followed by scoring the suppression or enhancement of the previous eye phenotypes, can establish if both candidate genes interact genetically to regulate eye development. We took the same approach.

**Figure 3:**
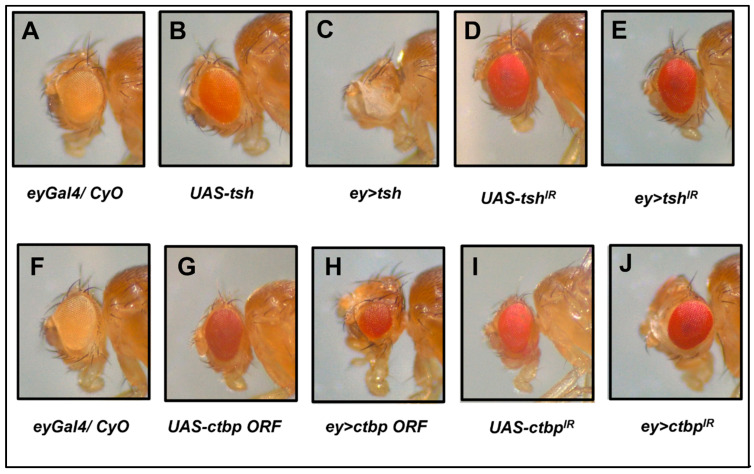
Light microscopy images of adult eyes of flies having up- and downregulation of *tsh* and *ctbp*, and respective controls. The adult eyes of the control flies, including the *eyeless-Gal4* (**A**,**F**) driver, and *UAS-tsh* (**B**); *UAS-tsh^IR^* (**D**); *UAS-ctbp ORF* (**G**); and *UAS-ctbp^IR^* (**I**) were compared with the adult eyes of their progenies (i) overexpressing *tsh* (*ey > tsh*) (**C**) and *ctbp* (*ey > ctbp ORF*) (**H**); and progenies having downregulation of (ii) *tsh* (*ey > tsh^IR^*) (**E**) or *ctbp* (*ey > ctbp^IR^*) (**J**) expression. The (**C**) *ey > tsh* flies lack the entire eyes or had tiny eyes; the (**E**) *ey > tsh^IR^* flies had no eye phenotype; the (**H**) *ey > ctbp ORF* flies had smaller eyes; and the (**J**) *ey > ctbp^IR^* flies had slightly bigger eyes relative to their parents’ eyes.

We crossed flies having the *eyeless-Gal4* (*eyGal4/ CyO*) (Figure 3A,F) driver with *UAS-tsh* (Figure 3B) and *UAS-ctbp ORF* (Figure 3G) flies to overexpress *tsh* or *ctbp* in the eye precursor cells of respective F1 progeny and record any change in their adult eye phenotypes relative to their parents. The F1 adult flies in which *eyGal4* was driving the overexpression of the *tsh* (*ey > tsh*) lacked both eyes or had tiny eyes with a few ommatidia (Figure 3C). The eye–antennal discs of the *ey > tsh* third instar larvae were either absent or very small without the eye disc part. Overexpression of *ctbp* by the same driver (*ey > ctbp ORF*) produced smaller eyes (Figure 3H), consistent with a previous report [22]. Next, we downregulated *tsh* or *ctbp* expression by the *eyGal4* driver and compared changes in the adult eye phenotype in these offspring with that of their parents [(*UAS-tsh^IR^*) and (*UAS-ctbp^IR^*)] (Figure 3D,I). Downregulation of *tsh* by *eyGal4* (*ey > tsh^IR^*) did not produce an obvious adult eye phenotype (Figure 3E), most likely because of the functional redundancy of its paralogue, Tiptop [45]. Downregulation of *ctbp* by *eyGal4* driver (*ey > ctbp^IR^*) produced slightly larger adult eyes (Figure 3J). These results confirmed that the spatio-temporal regulation of *tsh* and *ctbp* gene expression plays a major role during *Drosophila* adult eye development.

**Figure 4:**
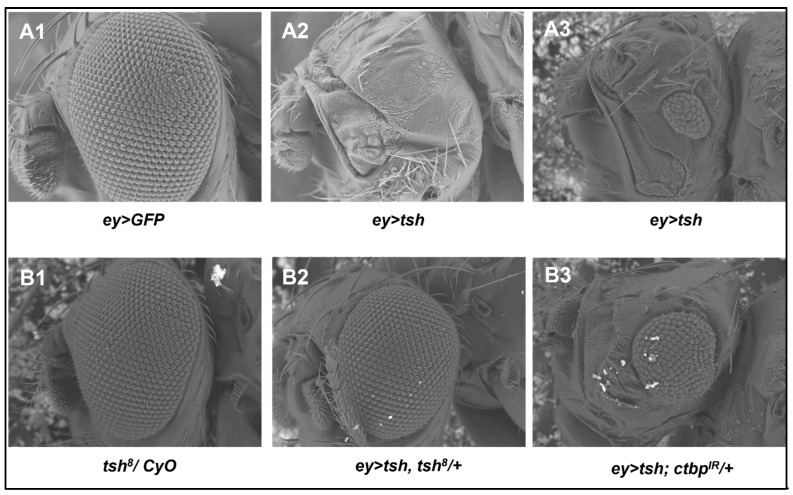
Scanning electron micrographs of adult fly eyes show that the genetic interaction of *tsh* with *ctbp* in the eye–antennal disc regulates eye development. Overexpression of *tsh* (**A2**,**A3**) but not *GFP* (**A1**) by the *ey-Gal4* driver produced no or tiny adult eyes. These no or tiny eye phenotypes are partially rescued in flies also having a **LoF** *tsh* allele (*tsh^8^*) (**B2**) or *ctbp* downregulation (**B3**). The control flies containing a **LoF** *tsh* allele (*tsh^8^/CyO*) (**B1**) or *UAS-ctbp^IR^* (Figure 3I) had normal adult eyes.

Genetic interaction between *tsh* and *ctbp* was shown during gut development [63], but such interaction is yet to be demonstrated in the context of *Drosophila* eye development. To investigate potential genetic interactions between *tsh* and *ctbp* and their impact on eye development, we conducted the next set of experiments. As discussed above, all adult flies in which *eyGal4* drove *tsh* overexpression (*ey > tsh*) had no eye (Figure 4(A2)) or tiny (with a few ommatidia) eyes (Figure 4(A3)) compared to the normal adult eyes present in the control flies (*ey > GFP*) (Figure 4(A1)). There is a LoF (Loss of Function) *tsh* allele (*tsh^8^*) containing a heterozygous fly strain (*tsh^8^/CyO*) that had normal adult eyes (Figure 4(B1)). The no eye or tiny eye phenotypes in the *ey > tsh* flies were partially rescued (~80% of wild type eye size) by introducing one LoF *tsh^8^* allele (*ey > tsh*, *tsh^8^/+*) (Figure 4(B2)), confirming that the absence of adult eyes in the *ey > tsh* flies was caused by the overexpression of *tsh*. Interestingly, the lack of eye or the tiny eye phenotype in the *ey > tsh* fly was partially rescued (~30 to 50% of wild-type eye size) by simultaneously downregulating *ctbp* expression (*ey > tsh; ctbp^IR^/+*) (Figure 4(B3)).

**Figure 5:**
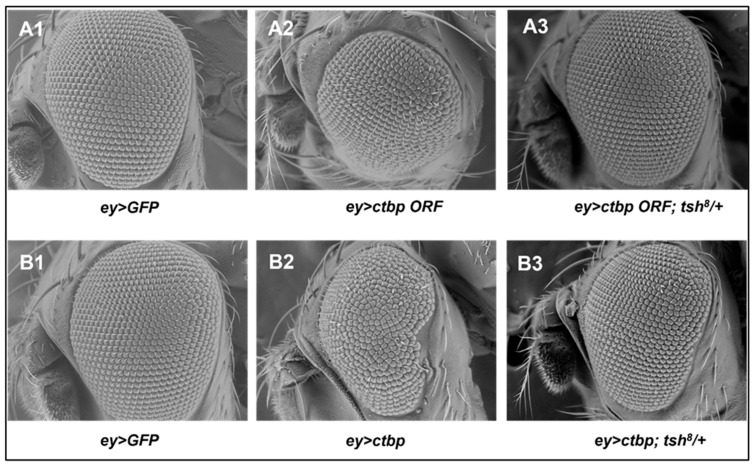
Scanning electron micrograph showing that the small or misshapen adult eye phenotypes generated by overexpression of *ctbp* were rescued by removing a functional allele of *tsh*. Overexpression of *ctbp ORF* (**A2**) but not *GFP* (**A1**) by the *ey-Gal4* driver produced small adult eyes (**A2**). The small eye phenotype was rescued in the flies also having a **LoF** *tsh* allele (*tsh^8^*) (**A3**). Overexpression of *ctbp* (**B2**) (a different *UAS-ctbp* line with the *P[GSV]^A396^*) but not *GFP* (**B1**) by the *ey-Gal4* driver produced adult eyes with lost ommatidia at the edges (**A2**). The absence of ommatidia at the edges was rescued in the flies also having a **LoF** *tsh* allele (*tsh^8^*) (**B3**). The control flies containing a **LoF** *tsh* allele (*tsh^8^*) (**B1**) or *P[GSV]^A396^* [22] had normal eyes.

Next, we performed converse experiments. The small eye phenotype in the flies overexpressing *ctbp ORF* by the *eyGal4* driver (*ey > ctbp ORF*) (Figure 5(A2)) compared to the control (*ey > GFP*) (Figure 5(A1)) was entirely rescued by one **LoF** *tsh^8^* allele (*ey > ctbp ORF*; *tsh^8^/+*) (Figure 5(A3)). When *eyGal4* drove *ctbp* (*ey > ctbp*) expression in a fly strain with a different *UAS-ctbp* construct (*P[GSV]^A396^*), it resulted in adult fly eyes with few missing ommatidia at the margin of the eye (Figure 5(B2)) that were present in the control eyes (*ey > GFP*) (Figure 5(B1)). The abnormal eye phenotype was suppressed by introducing one *tsh^8^* allele (*ey > ctbp; tsh^8^/+*) (Figure 5(B3)). These experiments were performed several times to establish the functional relationship between *ctbp* and *tsh* in the context of eye development, and we observed the same outcomes. Our experimental data (please check the Supplementary File 1 for sample number/genotype observed and quantification of ommatidia) imply that Tsh interacts with CtBP during eye development.

### 3.3. Tsh Associates with CtBP in the Third Instar Eye–Antennal Disc in Vivo

**Figure 6:**
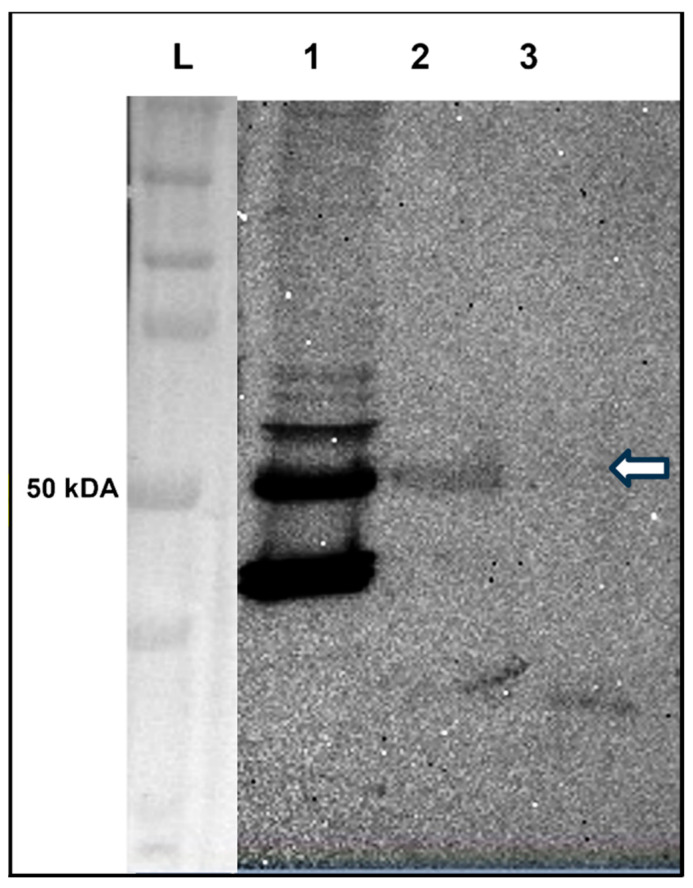
Co-immunoprecipitation showing Tsh pulls down CtBP from the lysate prepared from the third instar larval eye–antennal discs of *EGFP-tsh* transgenic flies. IP with anti-EGFP antibody and Western blot with anti-CtBP antibody using lysate prepared from third instar larval eye–antennal discs dissected from *EGFP-tsh* transgenic flies. The lane marked by “L” contains the protein ladders (BioRad Precision Plus Protein Dual Color Standards). A 50 kilo-Dalton (kDA) band representing CtBP was present in the crude lysate (lane 1). A similar-sized band was precipitated by anti-EGFP antibody (lane 2) but not by the Protein G beads (negative control) (lane 3). The white arrow marks the 50kDA band size.

Co-immunoprecipitation (Co-IP) is a trusted in vivo method to identify molecular interactions between proteins in their native forms within a biological sample. Transcription factors of RDGN, such as Dac, Dan, and Danr, which contain a PXDLS motif, have been shown to interact with CtBP in vivo and in vitro [22,28]. Tsh contains the PLDLS motif and has been shown to bind with CtBP by co-immunoprecipitation using a lysate prepared from the Kc167 cells [45]. Therefore, we performed Co-IP using lysates prepared from the third instar larval eye–antennal discs of *EGFP-tsh* transgenic flies. We used an anti-GFP antibody to precipitate EGFP-Tsh and detect CtBP in the precipitated complex using an anti-CtBP antibody. A 50 kDa band was detected with the anti-CtBP antibody in an immuno-precipitate obtained with anti-GFP antibody, but not in control (precipitate obtained with the protein G beads only, without any anti-GFP antibody) on Western blot (Figure 6). The 50 kDa band corresponds to the long isoform of CtBP [72] that was also detected in the crude lysate made from the eye–antennal disc dissected from the EGFP-tsh fly strain (Figure 6). Thus, Tsh and CtBP were both present in a molecular complex made from the eye–antennal disc, suggesting they interact directly at the molecular level during *Drosophila* adult eye development.

### 3.4. Tsh Can Directly Bind to CtBP in Vitro

**Figure 7:**
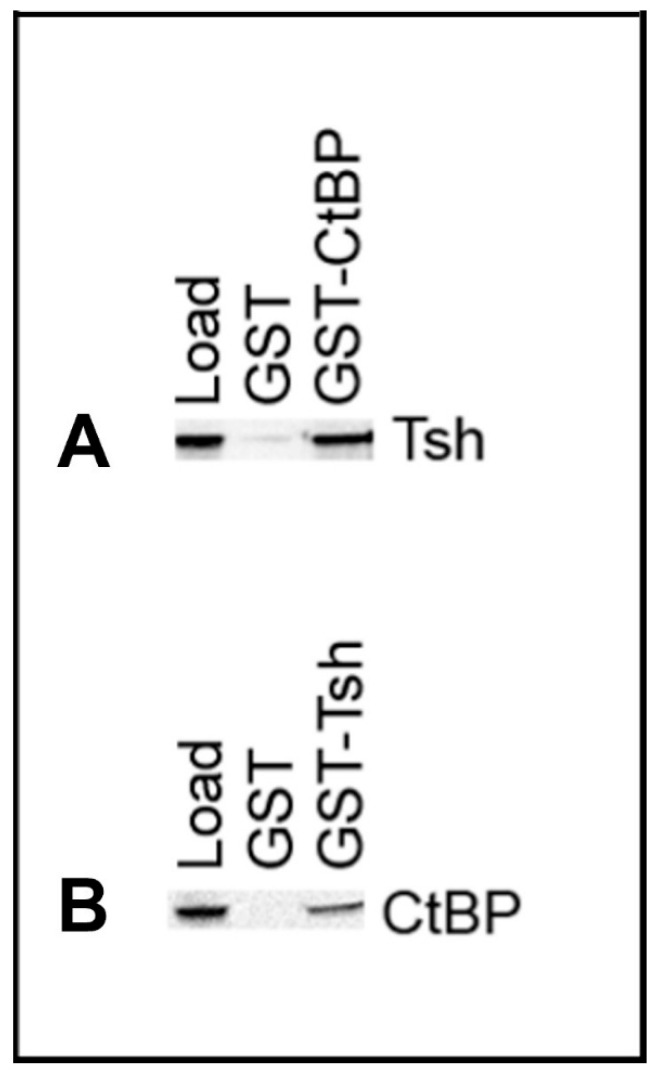
GST pulldown experiments reveal that Tsh and CtBP proteins bind directly. (**A**) shows that the GST-CtBP, but not the GST protein, can pull down the Tsh protein. The first lane contains the 10% sample from the in vitro translated Tsh prey protein band marked as “Load”, and the protein band in the third lane represents Tsh, pulled down by the GST-CtBP bait protein. The absence of the same band in the center lane signifies that GST alone could not pull down Tsh, suggesting CtBP binds and pulls down Tsh. (**B**) shows that the GST-Tsh, but not the GST protein, can pull down the CtBP protein. The first lane contains the 10% sample from the in vitro translated CtBP prey protein band marked as “Load”, and the protein band in the third lane represents CtBP, pulled down by the GST-Tsh bait protein. The absence of the same band in the center lane signifies that GST alone could not pull down CtBP, suggesting Tsh binds and pulls down CtBP.

The co-immunoprecipitation results prompted us to test if Tsh binds physically with the CtBP protein in the eye–antennal disc. We performed GST pulldown experiments to test this. We incubated in vitro translated and biotinylated Tsh or CtBP proteins with GST, GST-CtBP, or GST-Tsh. The biotinylated Tsh was pulled down by GST-CtBP but not by GST alone (negative control) (Figure 7A), and the biotinylated CtBP was pulled down by GST-Tsh but not by GST alone (negative control) (Figure 7B) as detected in Western blots. Thus, Tsh binds directly to CtBP.

## 4. Discussion

### 4.1. CRISPR and BAC Recombineering Are Useful Techniques to Produce Functional Tagged Proteins from Tagged Genes in the Genome

The use of immunohistological staining to visualize a protein’s expression pattern in a tissue and within cells, and the use of co-immunoprecipitation plus Western blot to identify protein-protein interactions, are common in cell and molecular biology research. One limitation for both techniques is the unavailability of antibodies. We also did not have anti-Tsh and anti-CtBP antibodies at the beginning of this project, which restricted us from performing the two techniques. So, we applied the strategy to tag our genes of interest, *tsh* and *ctbp*, in vivo with *GFP* and *FLAG* at their 5′ end, respectively, as antibodies against the tagged proteins GFP and FLAG are commercially available. Tagging a gene in the genome is very useful as it allows all the regulatory sequences of the gene to remain unaltered, such that the fused protein is expressed in its wild-type spatio-temporal pattern and amount. We successfully tagged the genomic *tsh* gene with an *EGFP* encoding gene by CRISPR-Cas9 mediated homology repair technique, and the *ctbp* gene with a *FLAG* encoding gene by BAC recombineering technique. We confirmed that both EGFP-Tsh and FLAG-CTBP proteins are functional, and both show their native expression pattern in the eye–antennal discs in histological staining performed using anti-GFP and anti-FLAG antibodies. The EGFP-Tsh transgenic flies later allow us to perform a Co-IP assay using an anti-GFP antibody.

### 4.2. Effect of Tsh and CtBP Interaction on Eye Development Varies with Their Relative Dosage

During *Drosophila* eye development, transcriptional activation and repression of eye specification genes depend on the interactions between different transcription factors and cofactors. These transcription regulatory molecules form distinct complexes in a spatio-temporal manner in the eye imaginal disc to produce eye precursor cells through cell division first, then, under the influence of other complexes, a different subset of genes is activated, which inhibit cell division and initiate differentiation to produce retinal cells [19,20]. Furthermore, different amounts of these factors can trigger feedback loops, which can further regulate their expression and the expression of their target genes, making the eye development mechanism highly complex [19,33,46,73].

It was reported that overexpression of different eye-specific transcription factors during early stages of development disrupted normal head and eye development in fruit flies [33]. To our knowledge, this is the first report revealing that overexpression of *tsh* by *eyGal4* driver (*ey > tsh*) severely represses eye disc and adult eye formation. We find that *ey > tsh* flies have antennal discs but hardly any visible eye discs. Thus, overexpression of *tsh* results in remarkable undergrowth of the eye disc, ultimately producing adult flies with no or tiny eyes. This loss of adult eye phenotype is rescued by removing a functional *tsh* allele [in other words, by adding one *tsh* null allele (*ey > tsh, tsh^8^/+*)], confirming that the *tsh* overexpression indeed caused the no (or very tiny) eye disc, and lack of (or tiny) adult eye phenotype. As described above, Ey and Tsh expression is detected as early as the first instar eye–antennal disc [20,23,29,30,47]. Thus, excess expression of *tsh* by the *eyGal4* driver in the early developmental stage of the eye–antennal disc hinders the growth of the eye disc and generates eyeless adult flies. Next, this no or tiny eye phenotype is suppressed partially by reducing *ctbp* expression in the same flies with *ctbp RNAi* expression induced by the same driver (*ey > tsh; ctbp^IR^/+*). As a result, the loss of eye or tiny eye phenotype is partially rescued. It is noteworthy that we have observed variability in the rescued eye size, which may be dependent on some degree of variation in the overexpressed *tsh* and downregulated *ctbp* amounts in individual flies. This genetic interaction suggests that Tsh interacts with CtBP during eye development. The results also suggest that the inhibition of eye–antennal disc growth by high doses of Tsh is achieved through its interaction with the wild-type amount of CtBP, because the strength of inhibition of eye-development by Tsh-gain of function is reduced with the downregulation of *ctbp* expression.

We performed converse experiments that also support the conclusion that Tsh and CtBP interact genetically during eye development. First, we overexpress *ctbp* by the *eyGal4* driver (*ey > ctbp ORF*), which produces smaller eyes as reported earlier [22]. The smaller eye phenotype has been rescued completely by removing a functional allele of *tsh* [in other words, by adding a null *tsh* allele (*ey > ctbp ORF; tsh^8^/+*)]. Earlier, it was suggested that CtBP inhibits eye precursor cell proliferation anterior to the MF [22]. Thus, *ctbp* overexpression is likely to produce fewer eye precursor cells, which ultimately results in producing adult eyes with fewer ommatidia. This eye repressor ability of CtBP depends on the amount of Tsh, because reducing the amount of Tsh also reduces the strength of this repression.

In summary, interaction between the wild-type amounts of Tsh and CtBP is required for normal eye disc and adult eye development in *Drosophila.* Excess expression of either *tsh* or *ctbp* inhibits adult eye development by reducing the number of eye precursor cells in the eye disc, which could be partially rescued by downregulating the amounts of either *ctbp* or *tsh,* respectively. Overall, Tsh and CtBP interaction limit the proliferation of eye precursor cells in the eye disc.

### 4.3. Both Coimmunoprecipitation and GST Pulldown Assays Provide Evidence for Tsh and CtBP Molecular Interaction in the Eye–Antennal Disc

Our study reveals for the first time that Tsh and CtBP interact with each other in the eye–antennal disc. Moreover, GST pulldown assays confirm that Tsh and CtBP bind directly to each other. We found that Ey and CtBP were found in a Co-IP but could not bind directly in GST pulldown assays [22], and that Ey and Tsh were present in a Co-IP [20]; thus, our results increase the likelihood that Tsh may work as a linker molecule to connect Ey and CtBP, which altogether limit the proliferation of eye precursor cells anterior to the furrow.

## 5. Conclusions

In conclusion, we present new findings that provide mechanistic insights into Tsh and CtBP interaction in controlling *Drosophila* eye development. This is the first report to provide conclusive genetic and molecular evidence that Tsh and CtBP bind directly in the *Drosophila* third instar larval eye–antennal disc, and that their interaction is required for the development of the normal adult eye. This work reveals novel information that ectopic expression of *tsh* by the *eyGal4* driver results in total or almost total loss of the eye–antennal disc and adult eye, which can be restored by reducing *ctbp* expression. Accordingly, the small adult eye phenotype caused by overexpression of the *ctbp* is restored by a *tsh* null allele. Our analysis indicates that the stoichiometry of these two proteins determines the outcome of their interaction on the eye development process. We propose that the interaction between Tsh and CtBP regulates eye precursor cell proliferation during the early stages of eye–antennal disc development. It will be interesting to identify the cellular response due to the manipulation of these genes’ expression and their interaction to elucidate the complete molecular mechanism exerted by Tsh and CtBP interaction in the future.

## Figures and Tables

**Table 1:** Different combinations of bait and prey proteins to perform GST pulldown experiments.

Combinations	Bait Protein	Prey Protein
A. This combination was used to verify if the GST-CtBP, but not the GST (control), could pull down Tsh in vitro.	GST-CtBP	Tsh
	GST	Tsh
B. This combination was used to verify if the GST-Tsh, but not the GST (control), could pull down CtBP in vitro.	GST-Tsh	CtBP
	GST	CtBP

## Data Availability

The original contributions presented in this study are included in the article/supplementary material. Further inquiries can be directed to the corresponding author(s).

## Supplementary Materials

The following supporting data file 1 (an Excel file with count of adult eye sample numbers per genotype, and average number of ommatidia in adult eyes per genotype) can be downloaded at: https://www.mdpi.com/article/10.3390/genes16091045/s1.
